# Primary care scribes: writing a new story for safety net clinics

**DOI:** 10.1136/bmjoq-2017-000124

**Published:** 2017-10-25

**Authors:** Christina Lowry, Katherine Orr, Brett Embry, Michael Nguyen, Amy Petersen, Catherine James, Keith Seidel, Neda Ratanawongsa

**Affiliations:** 1 Primary Care, San Francisco Health Network, San Francisco Department of Public Health, San Francisco, California, USA; 2 Division of General Internal Medicine, Department of Medicine, UCSF Center for Vulnerable Populations, University of California, San Francisco, San Francisco, California, USA

**Keywords:** electronic health records, primary health care, safety-net providers, workflow, patient satisfaction, workers, volunteer, efficiency, organizational

## Abstract

The spread of electronic health records systems (EHRs) poses challenges for both patient and provider care experience. Limited research suggests that scribes offer potential benefits to productivity and clinician satisfaction in emergency health and specialty settings. We conducted this evaluation of trained volunteer scribes for primary care clinics serving a diverse, low-income population in a US safety net system, which implemented a new EHR 2011–2014. The scribe programme trained and managed scribes for 51 providers (25% participation) from 5 of 12 San Francisco Health Network primary care clinics. We evaluated the programme using four measures. Providers reported spending less time out of clinic completing notes after sessions with scribes versus sessions without scribes (14.0 min vs 30.2 min, p<0.01). The rate of incomplete EHR notes at 72 hours was not significantly different for clinics using and not using scribes (16.9% vs 16.7%, p=0.4). Mean visit length using EHR-recorded provider cycle time was shorter for sessions with scribes (24.0 min), compared with sessions without scribes (26.4 min, p<0.01). Patients at clinics using scribes were as likely to recommend their provider (74.5%), compared with patients at clinics not using scribes (74.3%). Limitations of our evaluation include selection bias and possible confounding by clinic- and provider-level factors. In a safety net primary care system, trained volunteer scribes were associated with improved clinician efficiency and experience and no difference in patient satisfaction.

## Problem

Electronic health records (EHRs) have spread rapidly in the USA with federal incentives triggered by the 2009 Health Information Technology for Economic and Clinical Health Act, which enabled resource-limited safety net health systems to afford fully functional EHRs for the first time.^1^ However, early research suggests that these newer certified EHRs are associated with higher clerical burdens for providers, who report higher rates of stress and burnout.^2 3^ The impact of EHR implementation may be particularly challenging in safety net clinics serving diverse, complex patient populations, where providers may be at risk for lower professional satisfaction.^2 3^

San Francisco Health Network (SFHN) primary care clinics provide over 290 000 visits per year to a low-income population composed of 35% Latino, 25% Asian, 17% African-American and 17% White patients.^4^ Its mission is to provide high quality, comprehensive, culturally proficient health services and to ensure equal access to all.^4^ From 2011 to 2014, to advance this quality of care, SFHN primary care clinics implemented a new EHR certified to meet US Centres for Medicare and Medicaid Services incentives. However, after EHR implementation, SFHN providers reported stress, increased in-clinic and after-hours charting, and decreased satisfaction with their clinical relationships and professional work.

In response, SFHN implemented a primary care scribe programme to reduce the clerical burden of EHRs, improve the rates of timely completion of EHR notes and improve the care experience of providers and patients, while offering future medical professionals a mentoring environment within safety net health systems. By the end of 1 year, the programme aimed to

- reduce provider-reported time out of clinic completing notes to 20 min,
- decrease the rate of incomplete EHR notes at 72 hours to 10%,
- reduce the mean visit length by 2 min,
- maintain or increase the proportion of patients who would recommend their provider.

## Background

Since the implementation of newer EHRs, providers have experienced a shift and increase in clerical burdens during and after clinical hours.^2 3^ In onetime-motion study, providers allocated 50% of their time in the office to electronic documentation, 27% face-to-face time with patients and approximately 2 hours of time outside of clinical sessions completing EHRs.^2^ A disproportionate amount of time spent on documentation can contribute to provider burnout and decreases retention.^5^ A US survey found that physicians who used EHRs were less satisfied with the amount of time spent on clerical tasks and were at higher risk for professional burnout.^3^

The medical scribe industry has grown rapidly to meet the documentation challenges of EHRs.^6^ Medical scribes are unlicensed individuals who enter information into the EHR under clinician supervision in real time.^6^

Numerous studies have examined the positive impact of medical scribes in emergency health and specialty settings worldwide.^7–10^ Medical scribes have been associated with increased provider productivity, enabling providers to see more patients.^8^ Other studies report that scribes are associated with increased provider satisfaction, patient flow and organisational revenue.^9^ This research suggests that medical scribes allow providers to prioritise and complete their clinical duties in a more efficient manner.^7–10^

Thus, SFHN invested in a small number of centrally funded scribe coordinators, who trained and managed unpaid volunteer scribes for 50 providers (25% participation) from 5 of 12 SFHN primary care clinics using a certified EHR through a competitive volunteering programme, attractive to both clinics and volunteers alike.

## Measurement

We selected four metrics with a control group for each (table 1): (1) provider-reported time out of clinic completing notes, (2) rate of incomplete EHR notes, (3) mean visit length using EHR provider cycle time and (4) patient satisfaction on patient survey.

**Table 1:** Metrics for evaluating a medical scribe programme for safety primary care providers

Metric	Intervention group	Control group	Source	Data collection period	Limitation
Time spent completing medical notes outside of clinic	Provider sessions with scribes at five clinics with scribe programme	Provider sessions without scribes at five clinics with scribe programme	Daily provider survey	July 2016–December 2016	Self-report and recall bias
Incomplete notes at 72 hours	Clinics with scribes	Clinics without scribes	EHR-generated incomplete notes report	January 2016–November 2016	Intervention group data includes sessions and visits without scribes. This analysis excluded hospital-based clinic where aggregated data combined providers who did and did not use scribes.
Visit length with provider	Provider sessions with scribes at five clinics with scribe programme	Provider sessions without scribes at five clinics with scribe programme	EHR-generated provider cycle time report	January 2016–November 2016	At one clinic, scribes were used only for visit types for complex patients, new patients or for providers with longer cycle times. Scribes were among staff who recorded patient visit status in the EHR during scribed visits. This analysis excluded the hospital-based clinic where providers with scribes used them for all sessions.
Patient satisfaction	Patients receiving primary care from clinics with scribes	Patients receiving primary care from clinics without scribes	Clinician and Group Consumer Assessment of Healthcare Providers and Systems survey	January 2016–November 2016	Low response rate, limited to English-speaking and Spanish-speaking patients. This analysis excluded hospital-based clinic where aggregated data combined providers who did and did not use scribes.

### Provider-reported time out of clinic completing notes

Among providers using scribes, we conducted a daily written survey asking whether they used a scribe during the previous session and how many minutes they spent completing notes for the previous clinic session. We compared the average out of clinic time spent completing notes for sessions with a scribe and sessions without a scribe.

### Incomplete notes at 72 hours

Operational leadership runs a weekly report on the number of notes that remain incomplete in the EHR at 72 hours after the visit. In addition to serving as a marker of provider efficiency, incomplete notes can have negative impacts on clinical care because of missing clinical documentation and on financial sustainability because they are required to bill insurance for services rendered. Each week, we averaged the number of incomplete notes at each of the 12 primary care clinics. We compared this average for the five clinics with scribes to the seven clinics without scribes.

### Visit length

In clinics using scribes, operational leadership reports provider cycle time based on the status of a patient throughout the steps of the visit from registration through discharge, as recorded in the EHR by clinic staff (including scribes). We compared mean session provider cycle times for half-day sessions between sessions using scribes and sessions not using scribes. As a secondary analysis, among providers using scribes, we also calculated how often the sessions with scribes had shorter visit lengths than sessions without scribes.

### Patient Satisfaction

English-speaking and Spanish-speaking patients who receive primary care receive mailed Clinician and Group Consumer Assessment of Healthcare Providers and Systems (CG-CAHPS) visit survey.^11^ For this evaluation, we focused on the overall satisfaction measure: ‘Would you recommend this provider’s office to your family and friends?’ We categorised answers as ‘Yes, definitely’ versus ‘Yes, somewhat’ or ‘No.’ San Francisco Department of Public Health (SFDPH) operational leadership uses a 3-month rolling average for this data for each clinic to allow for late return of surveys and increase the sample size for each month’s report. We compared average satisfaction for clinics using scribes and clinics not using scribes. For this metric, we excluded the hospital-based clinic using scribes since the aggregate data combined providers who did and did not use scribes.

Visit length and patient satisfaction were also balancing measures. In theory, the presence of scribes in the room could result in less efficient clinical workflows and lower patient satisfaction due to integration of a third party in the room, inhibiting disclosure of sensitive medical information.

The limitations of these evaluation measures are shown in table 1.

This evaluation received an ‘exempt’ status from the University California San Francisco Committee on Human Research (protocol #17–22017) since this involved an evaluation of a quality improvement programme.

## Design

Medical directors of the primary care clinics recognised that a drop in the morale and productivity of their providers after the implementation of EHR and approached the ambulatory EHR standards committee for permission to implement medical scribes in primary care. SFHN primary care and network leadership were amenable to using scribes if they could comply with regulatory guidelines for billing and documentation compliance. After reviewing existing guidelines and other institution’s policies, an interdisciplinary group of key stakeholders including clinic medical and nursing leadership, the network compliance officer, the health information management (medical records) leadership, medical education leadership and the chief medical informatics officer participated in drafting and editing a scribe documentation policy and procedures.

A group of champion medical directors developed a training and supervision plan for four part-time paid scribes, ensuring they had proficiencies in understanding medical terminology, navigating and documenting within the EHR, and complying with policy limits for roles and responsibilities. The integration of scribes into clinic flow was discussed with nursing and medical assistants at the two pilot clinic sites.

In September 2013, in the two pilot clinics, scribes accompanied providers as they entered visit rooms, introducing the scribes and describing their role to patients. Scribes then logged into the EHR and documented the history, medication and allergy reconciliation, physical examination, assessment and treatment plan as narrated by the patient and provider. Scribe entries contained language clearly indicating their entries, and providers reviewed and revised all scribe documentation and attested to their accuracy. On the basis of the experiences of the pilot clinics, the implementation team decided to scale up to additional sites. By July 2016, SFHN hired five hourly scribe coordinator positions to standardise and evaluate the scribe programme across five clinics.

The Scribe Leadership Team—composed of these coordinators, the Chief Medical Officer for primary care, champion medical directors, public health professionals, clinical staff, quality improvement team members and data analysts—met monthly to develop the metrics for programme evaluation and leading dissemination of outcomes to SFHN stakeholders.

## Strategy

Plan Do Study Act (PDSA) cycle 1 (September 2013–December 2015) involved a pilot of four scribes at community clinic 1 in September 2013, at community clinic 2 in July 2013 and a hospital-based community clinic in August 2014. Each clinic’s medical director trained volunteers in medical terminology and how to navigate the EHR. The physicians at each clinic managing the scribe cohorts spent on average 4–5 hours per week during the 1–2 months of on-boarding and training. They spent 2–4 hours each week after the initial months scheduling, training, debriefing with scribes and working with clinical staff to establish a scribe workflow. Small group debrief of participating providers and staff on primary care teams revealed high provider and staff satisfaction overall, but no objective data were recorded.

PDSA cycle 2 (January 2016–June 2016) involved scaling up the programme to five clinic sites. Champion medical directors estimated that coordinating schedules and supervising scribes required 1–4 hours per week. Finally, operational leadership requested rigorous evaluation metrics and needed on-site, real-time data collection.

For PDSA cycle 3 (July 2016–December 2016), five salaried scribe coordinators were hired to oversee 40 volunteer scribes. Scribe Coordinators recruited volunteers, conducted trainings, provided 1:1 evaluation and feedback, coordinated schedules, refined workflow, liaisoned with providers and the clinical leadership, and designed the programme evaluation.

Coordinators recruited volunteer scribes from area schools and post-baccalaureate health professional preparation programme. Candidates that demonstrated availability for 8 hours a week for 10–12 months, along with a strong academic profile and commitment to the urban underserved were selected for interviews. Standardised questions were asked to assess the volunteers’ commitment to the underserved, academic proficiency, familiarity with medical terminology, clinical experience and non-English language proficiencies. Potential candidates possessed more experience and linguistic diversity as this cycle progressed.

Coordinators facilitated 20–40 hours of initial training at community clinic 1, including simulated visits and actual visits, followed by additional training at their assigned clinics. Scribes demonstrated the following competencies by the end of training:

- Print educational materials, future appointments and discharge summary from EHR;
- Merge discharge instructions, applicable templates and previous assessments and plans;
- Import diagnoses from the problem list/assessment;
- Open lab results, diagnostic imaging results, patient docs, healthcare maintenance results and immunisation records for the provider to view;
- Complete required checkboxes and steps required for meaningful use attestation;
- Enter content for referrals to the behavioural health team;
- Update the EHR visit status and location of patient through the clinic flow;
- Record a well-organised history of present illness section capturing at least 90% of pertinent information;
- Update patient contact information in EHR.

Coordinators also educated providers and staff at each clinical site about the scribe integration into clinic workflow. Best practices for workflow included the following:

- Clear communication of the responsibilities for scribe and certified medical assistant within each clinic’s workflow;
- Post-visit check-ins between the scribe and physician to verify discharge instructions;
- Physicians and scribes logged into separate EHR sessions within one computer, allowing physicians to put enter orders during visits;
- Laptops available in case all computers in the general workspace were full.

During the evaluation period, 5 of the 12 SFHN primary care clinics were using scribes: four of the nine community clinics and one of the three hospital-based clinics. All primary care clinics were offered the opportunity to participate in the scribe programme. Clinic leadership, with provider and staff input, decided whether to participate in the scribe programme. Within each participating clinic, providers could opt to work with a scribe or not. A total of 37 providers (97%) at the community-based clinics chose to work with a scribe, and 14 hospital-based providers (58%) chose to work with scribes.

All providers who participated in the programme worked with scribes for all patients within a session, with rare exclusions based on patient preference. Providers with scribes used scribes for most clinic sessions, unless the scribes were unavailable due to illness or their primary school/job responsibilities. At one clinic (community clinic 2), scribes were prioritised for visit types expected to have complex patients and patients establishing care or for providers with longer cycle times. The hospital-based clinic providers used scribes for almost all clinic sessions.

## Results

Operational leadership lacked baseline measurements during the initial PDSA cycles, and so metrics were developed during PDSA cycle 3 to allow for a controlled evaluation across prior cycles, using operationally available data.

### Provider-reported time out of clinic completing notes (n=667 surveys, figure 1)

**Figure 1:**
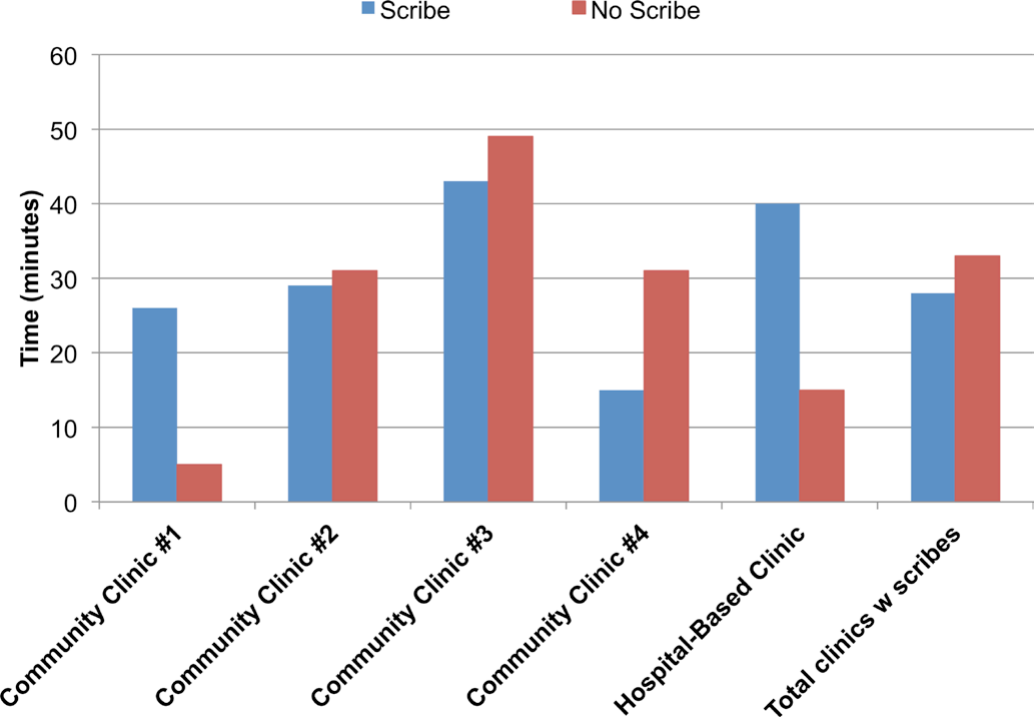
Average time spent completing medical notes outside of clinic for provider sessions with scribes versus sessions without scribes at five clinics with the scribe programme (n=667).

After sessions with scribes, providers reported an average of 14.0 min (SD 20.6) completing notes out of clinic versus 30.2 min (SD 26.0, p<0.01).

### Incomplete notes at 72 hours

**Figure 2:**
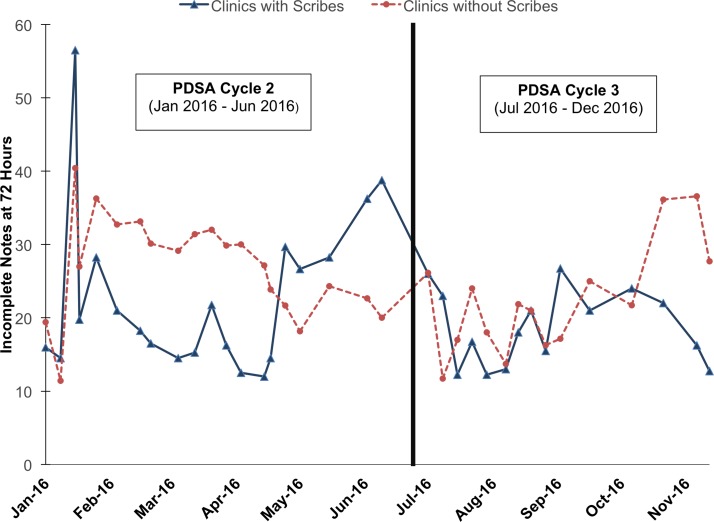
Incomplete notes at 72 hours comparing clinics with scribes versus clinics without scribes (January 2016–November 2016).

The rate of incomplete EHR notes at 72 hours was not significantly different for clinics using and not using scribes (16.9% (SD 4.1) vs 16.7% (SD 3.4), p=0.4). Both intervention and control clinics had an average of 15–18 incomplete notes per week. Figure 2 shows the absolute numbers of unlocked notes over PDSA cycles 2 and 3 at the clinics with and without scribes.

### Visit length (n=16 257)

The mean visit length for sessions with scribes was 24.0 min (SD 6.0) and for sessions without scribes to be 26.4 min (SD 6.1, p<0.01). Analysed another way, 70% of clinicians exhibited a faster cycle time when scribes were present. Data from the hospital-based clinic were not used because providers used scribes for almost all sessions so comparison data were not available.

### Patient satisfaction (n=5863, 27.0% response rate)

**Figure 3:**
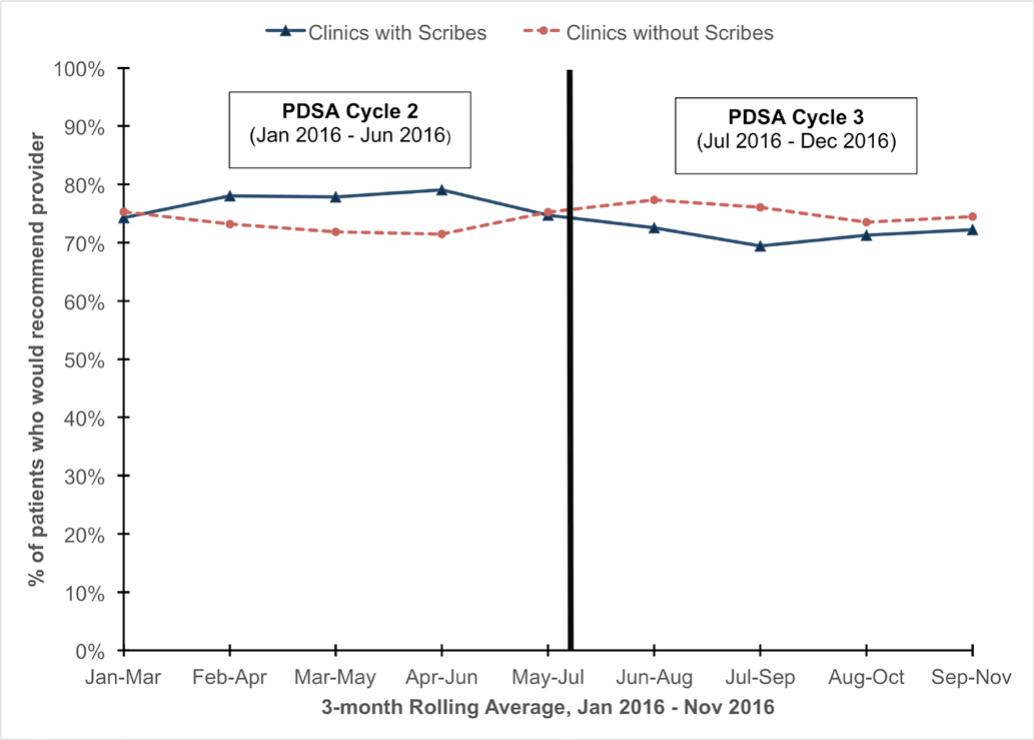
Patient satisfaction comparing patients receiving primary care from clinics with scribes versus patients receiving primary care from clinics without scribes (January 2016–November 2016, n=5863).

Among patients attending visits at clinics with scribes, 74.5% of patients would recommend their providers, compared with 74.3% (p>0.05) at clinics without scribes. Figure 3 shows the run chart of 3-month rolling average of patient satisfaction comparing clinics with scribes and clinics without scribes during the data capture period.

The limitations of the data and the control group comparisons are described in table 1.

## Lessons and limitations

The integration of unpaid volunteer scribes into safety net primary care offered a variety of challenges. First, we recruited and trained a cadre of unpaid volunteers and ensured that they had the necessary skills to serve in this role. As with any volunteer programme, high turnover could impair the quality of the programme, and so we requested that volunteers commit to 10–12 months in the position. While we were mostly successful in recruiting a dedicated group who commit to 8–10 hours per week and could be retained for 12 months, we had some turnover of scribes. Also, we were unable to meet our goal to recruit a large proportion of under-represented-in-medicine minority students to participate in the programme. For optimal recruitment and to minimise turnover, we recommend that volunteer cohorts be recruited during semesters and train during academic breaks.

Second, salaried positions are required to provide adequate training, supervision and coordination of a large group of volunteer scribes. The cost of the scribe programme at our five clinics included hourly salaries of US$22.50 for the lead scribe coordinator and US$18.50 for 2.5 full-time equivalent of other scribe coordinators. With benefits and support for professional development, the 22-month programme costs approximately US$150 000. While we did not conduct a cost-effectiveness analysis for this evaluation, we estimate this cost to be significantly lower than vendor-provided scribes, and thus the return on investment may be higher for resource-limited settings. Existing clinical leadership and staff did not have the bandwidth in safety net clinics to design and teach a curriculum including all of the EHR, medical and communication proficiencies required for medical scribes. This also requires flexibility from providers and staff as new recruits are beginning their training. As an academically-affiliated clinic system with multiple training programmes, SFHN had a culture of integrating team members into the clinical workflow and examination room. We recommend allocating resources to provide on-site coordinators for any volunteer scribe programme. In addition, scribe training curriculum in a safety net system should include information and medical terminology related to common health and social conditions that affect underserved communities. Future evaluations should examine cost-effectiveness of programme such as these.

Third, the training clinic, community clinic 1, was burdened by the responsibility of providing clinical training during patient care for all new scribes. While this model helped standardise the training experience to ensure proficiency for each new scribe, an alternative model of training new scribes at their future clinic would reduce the burden on one clinic. This would increase the responsibilities for the on-site coordinators.

The evaluation of the scribe programme was also challenging, leaving open the possibility of bias in each analysis. Because our EHR lacked out-of-the-box data analytics to compare note efficiency and visit length, operational leadership needed to develop these metrics after the scribe programme was initiated. Clinics and providers self-selected into programme participation, so confounding factors at both levels may have affected our comparisons. Providers who feel comfortable with EHR documentation are less likely to elect to use scribes, which likely led to conservative results in the incomplete note and visit length metrics.

Our analysis also did not permit our ability to investigate differences in the evaluation by clinic site, and future evaluation should include qualitative evaluation to explore the clinic and programme factors that promoted greater success in certain sites.

Both the patient and provider survey metrics are subject to recall bias and selection bias. The provider survey was only administered in clinics where scribes were used, preventing comparison with out-of-clinic documentation time in clinics without scribes.

Finally, the CG-CAHPS survey is only available in English and Spanish. Its items did not specifically focus on the care experience as related to scribes, and other confounding factors may explain the small difference in satisfaction measured. Future evaluations should be tailored to diverse populations to elicit their comfort with scribes in the room and their perceptions of the impact of scribes on the quality of patient-provider communication.

## Conclusion

Our evaluation contributes to the emerging literature about scribes by offering information about the standardisation of training and implementation of volunteer scribes in primary care in a safety net health system serving a linguistically diverse population. With multidisciplinary input, we developed a rigorous programme to coordinate the recruitment, training and optimisation of volunteer scribes. Our evaluation suggests the potential for volunteer scribes to improve clinical productivity as well as both provider experience, while maintaining patient satisfaction. EHRs should provide analytical tools to permit automated capture of scribe use and analyse their impact on clinical documentation efficiency. Future studies should investigate how to identify providers who would most benefit from scribe documentation support and how to elicit from diverse patients the impact of scribes on their care experience. Finally, programmes like ours should continue to explore the potential for supporting a pipeline to develop a diverse healthcare workforce of future health professionals committed to safety net care.
